# A Novel Gene Prognostic Signature Based on Differential DNA Methylation in Breast Cancer

**DOI:** 10.3389/fgene.2021.742578

**Published:** 2021-12-08

**Authors:** Chunmei Zhu, Shuyuan Zhang, Di Liu, Qingqing Wang, Ningning Yang, Zhewen Zheng, Qiuji Wu, Yunfeng Zhou

**Affiliations:** ^1^ Department of Radiation and Medical Oncology, Zhongnan Hospital of Wuhan University, Wuhan, China; ^2^ Hubei Key Laboratory of Tumor Biological Behaviors, Zhongnan Hospital of Wuhan University, Wuhan, China; ^3^ Hubei Cancer Clinical Study Center, Zhongnan Hospital of Wuhan University, Wuhan, China

**Keywords:** DNA methylation, gene expression, breast cancer, prognostic signature, the cancer genome atlas (TCGA)

## Abstract

**Background:** DNA methylation played essential roles in regulating gene expression. The impact of DNA methylation status on the occurrence and development of cancers has been well demonstrated. However, little is known about its prognostic role in breast cancer (BC).

**Materials:** The Illumina Human Methylation450 array (450k array) data of BC was downloaded from the UCSC xena database. Transcriptomic data of BC was downloaded from the Cancer Genome Atlas (TCGA) database. Firstly, we used univariate and multivariate Cox regression analysis to screen out independent prognostic CpGs, and then we identified methylation-associated prognosis subgroups by consensus clustering. Next, a methylation prognostic model was developed using multivariate Cox analysis and was validated with the Illumina Human Methylation27 array (27k array) dataset of BC. We then screened out differentially expressed genes (DEGs) between methylation high-risk and low-risk groups and constructed a methylation-based gene prognostic signature. Further, we validated the gene signature with three subgroups of the TCGA-BRCA dataset and an external dataset GSE146558 from the Gene Expression Omnibus (GEO) database.

**Results:** We established a methylation prognostic signature and a methylation-based gene prognostic signature, and there was a close positive correlation between them. The gene prognostic signature involved six genes: *IRF2, KCNJ11, ZDHHC9, LRP11, PCMT1,* and *TMEM70.* We verified their expression in mRNA and protein levels in BC. Both methylation and methylation-based gene prognostic signatures showed good prognostic stratification ability. The AUC values of 3-years, 5-years overall survival (OS) were 0.737, 0.744 in the methylation signature and 0.725, 0.715 in the gene signature, respectively. In the validation groups, high-risk patients were confirmed to have poorer OS. The AUC values of 3 years were 0.757, 0.735, 0.733 in the three subgroups of TCGA dataset and 0.635 in GSE146558 dataset.

**Conclusion:** This study revealed the DNA methylation landscape and established promising methylation and methylation-based gene prognostic signatures that could serve as potential prognostic biomarkers and therapeutic targets.

## Introduction

Breast cancer (BC) is the most prevalent cancer and the leading cause of cancer mortality in women worldwide (Freddie et al., 2020). In the United States, it is estimated that around 13% of women will suffer from BC in their lifetime (DeSantis et al., 2019). In recent years, the mortality of BC has gradually declined and the 5-years OS rate has reaches 90% attributed to early detection and improved treatment (Allemani et al., 2018).

Breast cancers are categorized into ER+, ER+/HER2-, HER2+ and Triple-negative subtypes based on the expression of estrogen receptor (ER), progesterone receptor (PR) and human epidermal growth factor receptor 2 (HER2). Similarly, gene expression analysis of these receptors further recognizes four subsets of BC: luminal A, luminal B, HER2-enriched (HER2-E) and Basal-like (Parker et al., 2009). These classification systems not only help predict the prognosis of BC patients, but also guide treatment choices. Conventional therapies such as surgery, radiotherapy, and chemotherapy form the basis of BC treatment. In addition, endocrine therapy for hormone receptor-positive BC and anti-HER2 treatment for HER2 expressing BC have greatly improved the prognosis of patients. Unfortunately, triple negative breast cancer (TNBC) still lacks effective therapeutic targets. Recent studies demonstrated that poly ADP-ribose polymerase 1 (PARP1) inhibitors and immune checkpoint inhibitors (ICIs) showed potential effect in TNBC (Mittendorf et al., 2020), (Schmid et al., 2020). Despite the great achievements in treatment, about 25–40% of BC patients will develop metastases (Siegel et al., 20172017). Among them, bone metastases are the most common, and approximately 75% of late-stage BC patients are diagnosed with bone metastases (Tulotta and Ottewell, 2018). Moreover, 5–20% of BC patients would have brain metastases (Achrol et al., 2019). Once the patient develops metastasis, the prognosis is poor, with the median OS of only 1–2 years (Redig and McAllister, 2013), (Martin et al., 2017). Therefore, it is urgent to find potential prognosis-related biomarkers to accurately predict the prognosis of BC patients.

Epigenetics events such as DNA methylation, histone modifications, chromatin remodeling, and non-coding RNAs play essential roles in the regulation of gene expression and actively participate in the development and progression of cancers. DNA methylation, which affects gene expression without changing the DNA sequence, is the most common epigenetic modification (Nakao, 2001), (Strahl and Allis, 2000). Stefansson et al. demonstrated that abnormal methylation of CpG islands in the promoter regions might activate proto-oncogenes or silence tumor suppressor genes, thereby contributing to the occurrence and development of tumors (Stefansson and Esteller, 2013). Accumulating evidences showed that decreased levels of genome-wide methylation were a critical sign of early cancers and were related to cancer grade and metastasis (Yang and Schwartz, 2011), (Ding et al., 2019). Indeed, DNA methylation was associated with most malignancies including bladder cancer (Chen et al., 2020a), lung cancer (Liang et al., 2019), and gastrointestinal tumors (Woo et al., 2018), (Huang et al., 2018).

Emerging studies have revealed the important roles of DNA methylation in BC (Pasculli et al., 2018; Kresovich et al., 2019; Xu et al., 2020). For instance, distinct DNA methylation patterns and associated gene expression profiles were found in different molecular subtypes of BC. *SFRP1,* a tumor suppressor gene, was down-regulated by hypermethylation in ER + breast cancer, leading poor prognosis (Stefansson et al., 2015), (Park et al., 2012). Other genes such as *BRCA1*, *CDH1*, and *PTEN*, were also abnormally methylated in BC. These events could serve as potential therapeutic and prognostic biomarkers (FitzGerald et al., 1998; Pharoah et al., 2001; King et al., 2003; Walsh et al., 2006; Suijkerbuijk et al., 2008; Luo et al., 2016). However, the prognostic role of DNA methylation in BC remains incompletely demonstrated.

In this study, we used bioinformatics methods to determine the prognostic role of DNA methylation and constructed methylation-associated prognostic signatures for BC. This study will help unveil the significance of DNA methylation in BC and might help discover novel prognostic biomarkers.

## Materials and Methods

### Data Acquisition and Processing

**FIGURE 1:**
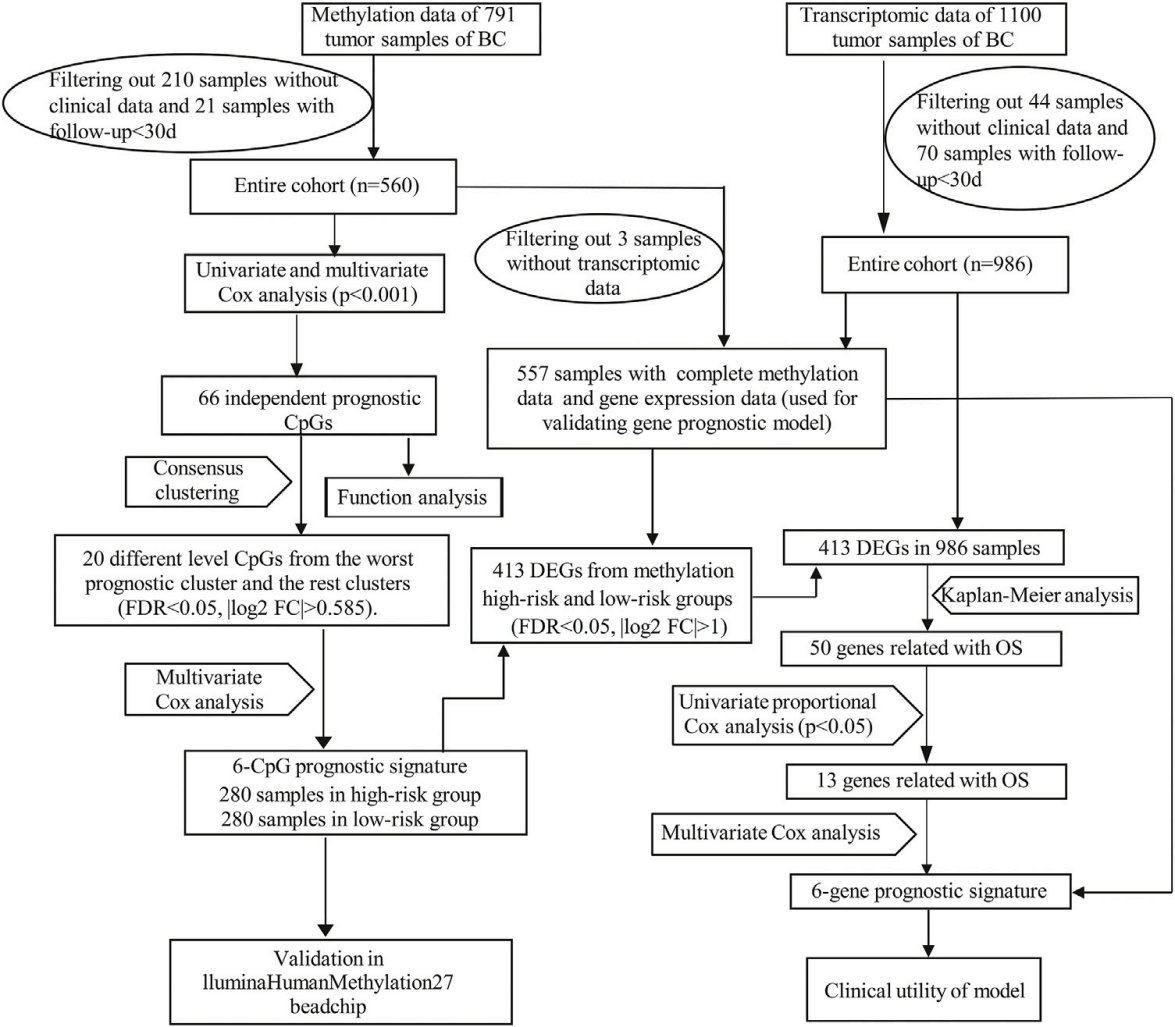
The workflow for this study. DEGs, differentially expressed genes.

RNA-seq data in fragments per kilobase of transcript per million mapped reads (FPKM) form and clinical information of BC were downloaded from the Cancer Genome Atlas (TCGA: https://portal.gdc.cancer.gov/) database. Illumina Human Methylation450 BeadChip array (450k array) and Illumina Human Methylation27 BeadChip array (27k array) data of TCGA database were downloaded from UCSC xena (https://xenabrowser.net/) (Goldman et al., 2020). DNA Methylation levels were evaluated by the β value, which ranged from 0 to 1 (0 means unmethylated and 1 means fully methylated). Probes with over 70% of missing values and probes located at chromosomes X and Y were removed. The missing values of the remaining probes were imputed using the k-nearest neighbours (knn) imputation algorithm of the impute R package. Since DNA methylation in promoter regions would strongly influence gene expression, we focused on the methylation probes in promoter regions defined as 2.0 kb upstream to 0.5 kb downstream from transcription start sites (TSS). Batch effects were removed by the ComBat algorithm of the sva R package (Leek et al., 2012). Ultimately, 560 patients including methylation data (from 450k array) and corresponding clinical data, 986 patients (from TCGA database) containing both gene expression data and corresponding clinical data were used for mainly analysis. And we obtained 557 overlapping patients (from the above two datasets) with complete gene expression data, methylation data and clinical data. Moreover, RNA-seq data and clinical information of 106 samples from GSE146558 were downloaded from NCBI (GEO: https://www.ncbi.nlm.nih.gov/) as external validation dataset. The mRNA expression profile from GEO dataset was normalized by the Robust Multichip Average (RMA) algorithm with background adjustment, quantile normalization, and final summarization. The workflow of our study was illustrated in Figure 1.

### Independent Prognostic CpG Loci Screening

Univariate Cox regression analysis was performed to screen out the prognosis-related CpGs of the 560 BC patients. In our study, we used the OS as clinical parameter of prognosis. Next, all these CpGs were subjected to multivariate Cox regression analysis, with age, pathological stage, and TNM stage as covariates to identify independent prognostic CpGs.

### GO and KEGG Analysis

The Gene Ontology (GO) and Kyoto Encyclopedia of Genes and Genomes (KEGG) (http://www.genome.jp/kegg/) (Kanehisa et al., 2017) analysis were performed using the R ClusterProfiler package (Yu et al., 2012). A *p* value < 0.05 was set as the cut-off value for both GO and KEGG analyses in our study.

### Consensus Clustering and Evaluation of CpG-Related Subtypes

Consensus clustering (Wilkerson and Hayes, 2010) was performed to determine subgroups of different methylation characteristics of the 560 BC patients based on the independent prognostic CpG loci using the ConsensusClusterPlus package of R. The criteria to determine the number of clusters were: (Freddie et al., 2020) The consistency within the cluster was relatively high; (DeSantis et al., 2019) There was no significant increase in the area under the CDF curve; (Allemani et al., 2018) The relative change in area under CDF curve tended to be stable. We then generated a consensus matrix to better visualize and help determine the number of clusters.

### Construction and Validation of the Methylation Prognostic Model

Differentially methylated independent prognostic CpG loci between the different prognosis clusters were screened out using Wilcoxon test. The filtering conditions were false discovery rate (FDR) < 0.05 and | log2 (fold change) | >0.585. On the basis of these differentially methylated CpG loci, a methylation prognostic model of 560 BC patients was constructed using multivariate Cox analysis. The formula of the risk score was as follows:

$$Risk\;score=\sum coef_{i}\beta_{i}$$

where coef_i_ was the multivariate Cox regression coefficient, and βi was the corresponding methylation β value. According to the median risk score (Chen et al., 2020b), (Shen et al., 2019), patients were divided into methylation low-risk (*n* = 280) and high-risk (*n* = 280) groups. Survival curves were employed to compare the OS of the two groups. Risk curve was plotted to visualize the relationship of the risk score, survival status and the methylated levels of the six signature CpG loci. Univariate and multivariate Cox regression analysis were performed to explore whether the risk score could be an independent predictor of OS. The sensitivity and specificity of the methylation prognostic model were evaluated by calculating the area under the curve (AUC) of the receiver operating characteristic (ROC) curve.

### Construction and Evaluation of Gene Prognostic Model

Differentially expressed genes (DEGs) were identified from methylation high-risk and low-risk groups (of 557 BC patients’ dataset) with the filter conditions of adjusted *p* < 0.05 and | log2 (fold change) | >1. And then we further extracted the above-mentioned DEGs from the transcriptomic profiles of 986 BC patients’ dataset for subsequent analysis. Kaplan-Meier analysis and univariate Cox regression analysis were employed to investigate prognosis-related DEGs of the 986 samples. Similarly, a gene prognostic signature was constructed by multivariate Cox regression analysis. And the risk formula was:

$$Risk\;score=\overset{n}{\underset{i=1}{\sum }}\left(expression_{i}\text{∗}coef_{i}\right)$$

Patients were also categorized into low-risk (*n* = 493) and high-risk (*n* = 493) groups based on the median risk score. Kaplan-Meier survival curve, risk curve, ROC curve, univariate and multivariate Cox regression analysis were also used for evaluating and validating the prognostic signature. Besides, two subgroups from 986 BC patients, the 557 patients’ dataset and GSE146558 dataset were used to validate the prognostic value of the gene signature.

### Antitumor Drug Sensitivity Analysis

CellMiner (https://discover.nci.nih.gov/cellminer/home.do) is a robust, user-friendly online database that integrates drug sensitivity and genomic data (Reinhold et al., 2017), (Wang et al., 2016). Anti-tumor activity data obtained from NCI-60 tumor cell line panel of the developmental therapeutics program (DTP) and RNA-seq data for the 60 cell lines of the NCI DTP drug screen were downloaded from this website. Subsequently, correlation between the sensitivity of anti-tumor drugs and the signature genes was analyzed.

### Statistical Analysis

R 3.6.3 (version 3.6.3, https://pan.baidu.com/s/1sufVf2lmoj9GYG_j5_fJKQ) was used for statistical analysis and plotting. Consensus clustering was performed using the ConsensusClusterPlus package of R; COX regression analysis was performed with the coxph function in survival package of R (Zhang, 2016); Kaplan-Meier curve was plotted using the survival and survminer packages of R; Pheatmap was plotted using the pheatmap package of R; The forest plots were plotted by the forestplot package of R; ROC curve was plotted by the survival ROC package of R. GO and KEGG analyses were performed using the ClusterProfiler package of R.

Mann-Whitney test was used to estimate the statistical significance of two groups of skewed distributed continuous variables, and Kruskal-Wallis test was used to evaluate the statistical significance of multiple groups of skewed distributed continuous variables (with Bonferroni correction for pairwise comparisons among multiple groups). All tests were two-sided and for all statistical tests, *p* < 0.05 was considered to be statistically significant unless otherwise specified.

## Results

### Screening of Prognosis-Related CpG Loci in Breast Cancer

**FIGURE 2:**
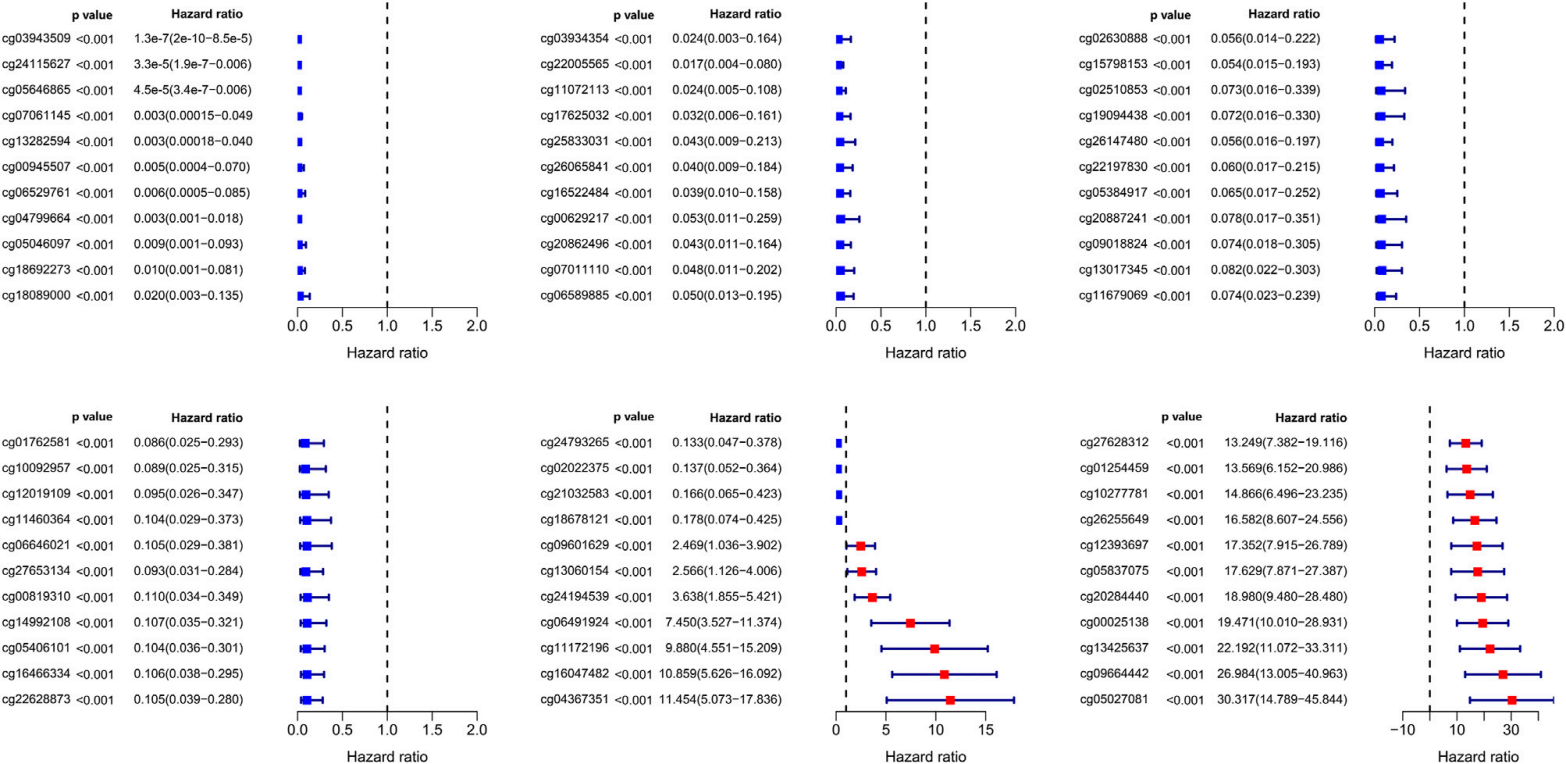
Significance and hazard ratio values of 66 independent prognosis-related CpG loci obtained from multivariate Cox regression analysis.

In our study, 450k array dataset was defined as train group and 27k array dataset was defined as test group (Table 1). Firstly, 144 CpG loci with *p* < 0.001 by univariate Cox analysis were screened out and identified as prognosis-related CpG loci. Using age, pathological staging and TNM staging as covariates, 66 CpG loci (49 CpG loci associated with favorable prognosis, 17 CpG loci associated with poor prognosis) with *p* < 0.001 by multivariate Cox analysis were further selected and used as the methylation classification features (Figure 2).

**TABLE 1:** General clinical characteristics of 560 BC patients.

Parameters	Methylation train group	Methylation test group
	(*n* = 560)	(*n* = 278)
Age	—	—
≤65 years	438 (78.3)	191 (68.7)
>65 years	122 (21.7)	87 (31.3)
Pathologic stage^a^	—	—
I/II	418 (74.6)	215 (77.3)
III/IV	142 (25.4)	57 (20.5)
unknow	0	6 (2.2)
T stage^a^	—	—
T1/2	479 (85.5)	238 (85.6)
T3/4	81 (14.5)	40 (14.4)
N stage^a^	—	—
N0	254 (45.4)	143 (51.4)
N1/3	306 (54.6)	128 (46.1)
unknow	0	7 (2.5)

a Staging according to Seventh Edition AJCC, Guidelines (Edge SB, Byrd DR, Compton CC, Fritz AG, Greene FL, Trotti A, eds. AJCC, Cancer Staging Manual. Seventh ed New York, NY: springer; 2010).

### Identification of DNA Methylation-Based Prognosis Subgroups

**FIGURE 3:**
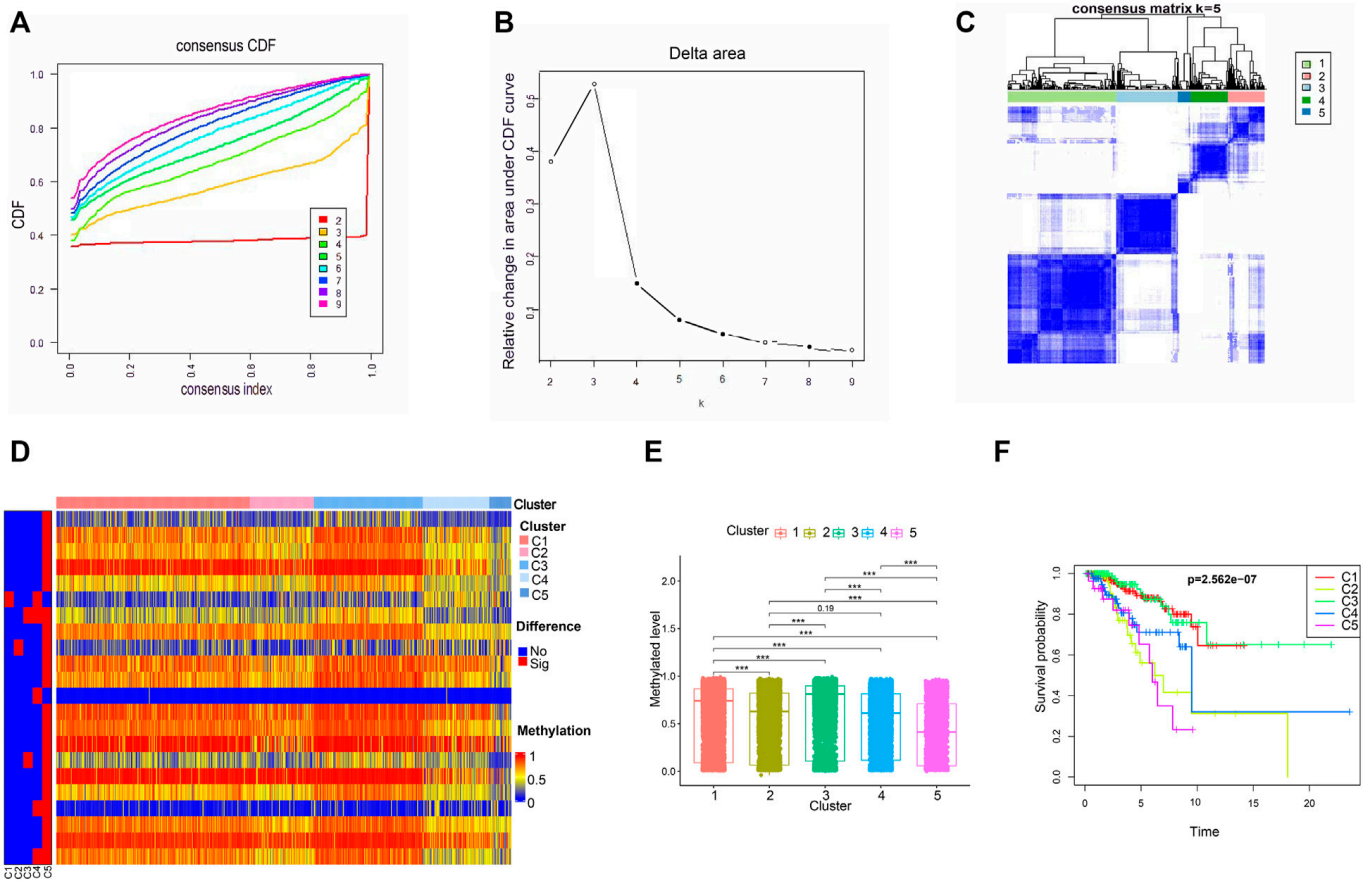
The methylated levels and prognosis of consensus clustering subgroups of breast cancer. **(A,B)** Consensus clustering cumulative distribution function for k = 2–9. **(C)** Consensus clustering matrix for k = 5. **(D)** Heatmap of the differentially methylated levels of the five subgroups. **(E)** Methylated levels of the 66 independent prognosis-related CpG loci among the five clusters. **(F)** Survival analysis of the five subgroups.

Then, the 560 patients were categorized into clusters of different methylation characteristics with consensus clustering based on the methylation of the 66 independent prognostic CpG loci. When the patients were assigned to 5 categories, the consistency within the clusters was high, the area under the cumulative distribution function (CDF) curve began to stabilize, and the relative change in area under CDF curve tended to be stable (Figures 3A,B). A consensus matrix representing the consensus for k = 5 also displayed a well-defined 5-block structure (Figure 3C). Accordingly, the optimal number of clusters was determined to be 5.

Subsequently, we conducted a subgroup analysis for the 5 clusters. Firstly, we compared the methylation levels of these 66 independent prognostic methylation loci among the five clusters. As illustrated in Figures 3D,E, Cluster 5 had the lowest methylation levels, followed by Clusters 2 and 4, Clusters 1 and 3. And the methylated difference between Cluster 5 and each of the remaining clusters was statistically significant. To explore the prognostic significance of the five clusters, Kaplan-Meier survival analysis was performed. We found that the prognosis was statistically significantly different among the 5 clusters, where Cluster 1 and Cluster 3 had the best OS, while Cluster 5 had the worst (Figure 3F).

### Construction and Evaluation of Methylation Prognosis Model

**FIGURE 4:**
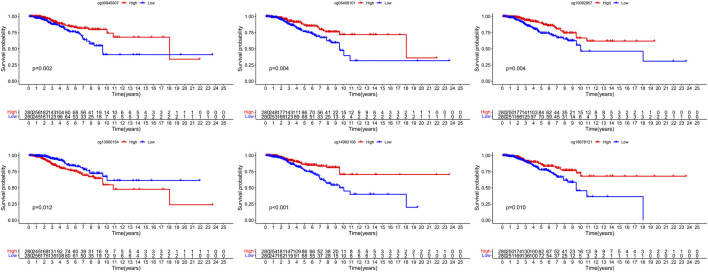
The survival difference of hypermethylation and hypomethylation of the six signature CpG loci.

The five clusters were significantly prognosis-associated, and therefore were used to identify potential prognostic biomarkers. Given that Cluster 5 had the lowest methylation level and the worst OS, it was reasonable to be selected as the reference cluster. Next, 20 differentially methylated independent prognostic CpG loci were identified from Cluster 5 and the rest clusters (Table 2). Ultimately, a methylation prognostic model was constructed which included six CpG loci (cg00945507, cg05406101, cg10092957, cg13060154, cg14992108, cg18678121) determined by multivariate Cox analysis (Table 3). Kaplan-Meier analysis showed that cg00945507, cg05406101, cg10092957, cg14992108, cg18678121 were associated with improved survival, and cg13060154 was associated with poor survival (Figure 4).

**TABLE 2:** Characteristics of the differential prognosis-related CpGs by wilcoxon rank-sum test (cluster 5 vs. the rest clusters).

CpGs	Log2 FC	*p* value	FDR
cg00945507	−1.078388031	6.76E-07	1.35E-06
cg02022375	−1.043706338	1.58E-12	1.30E-11
cg02630888	−0.851143204	1.04E-12	1.15E-11
cg05406101	−1.143837139	1.23E-15	8.12E-14
cg06646021	−0.804998375	5.45E-10	1.89E-09
cg10092957	−1.057286596	1.11E-07	2.44E-07
cg11072113	−0.594310951	1.86E-11	1.02E-10
cg13060154	0.6679634	0.030369072	0.04090528
cg14992108	−0.673767411	2.43E-10	1.07E-09
cg15798153	−0.959416408	2.90E-14	9.57E-13
cg16466334	−0.985246418	4.22E-12	3.09E-11
cg16522484	−0.696605561	9.22E-14	2.03E-12
cg18678121	−0.729884099	1.14E-07	2.44E-07
cg19094438	−1.478722276	5.50E-12	3.63E-11
cg21032583	−1.145936985	2.48E-13	3.28E-12
cg22197830	−1.050980669	1.22E-12	1.15E-11
cg24194539	1.144078012	0.010277521	0.014432263
cg26065841	−0.62551186	2.25E-13	3.28E-12
cg26147480	−0.695572224	1.37E-10	6.47E-10
cg27653134	−0.904508613	3.80E-11	1.93E-10

**TABLE 3:** Formula of Methylation prognostic model.

CpG loci	Coef	HR
cg00945507	−4.01607	0.018024
cg05406101	1.117929	3.058513
cg10092957	−1.78259	0.168202
cg13060154	1.655685	5.236664
cg14992108	−1.28305	0.27719
cg18678121	−1.1362	0.321038

Then we explored the mechanisms by which these signature CpG loci might act on BC. The six CpG loci, cg00945507, cg05406101, cg10092957, cg14992108, cg18678121, and cg13060154, were located at gene promoter regions of *SEC61G, RWDD2B, NCCRP1, SNTB1, SEC61A2, DAB2IP,* respectively. We firstly analyzed the correlation of these CpG loci and their corresponding target genes. The methylation of cg10092957, cg05406101, cg18678121, cg00945507 were moderately negatively correlated with the expression of their target genes. Whereas cg13060154 was weakly positively correlated with its corresponding gene (Supplementary Figures S1A–F). Consistent with the above results, the increased expression of *NCCRP1, RWDD2B, SEC61A2,* and *SEC61G* was associated with the decreased β values of cg10092957, cg05406101, cg18678121, and cg00945507, respectively. To the opposite of them, *DAB2IP* had higher expression in the presence of hypermethylated cg13060154 (Supplementary Figures S1G–L). However, there was no relationship between the cg14992108 and its target gene *SNTB1.* In addition, we took advantage of TCGA Wanderer, an interactive viewer exploring DNA methylation and gene expression data in human cancer (http://maplab.imppc.org/wanderer/) (Díez-Villanueva et al., 2015), to explore the methylated difference of the six CpG loci between breast cancer and normal tissues. We found that the methylation levels of cg05406101, cg18678121, cg14992108 were higher in normal tissues. However, the methylation levels of cg13060154 and cg10092957 were higher in breast cancer (Supplementary Figures S1M–R). We also explored the prognostic roles of the six CpG loci in breast cancer through the public database MethSurv (Modhukur et al., 2018) (https://biit.cs.ut.ee/methsurv/). High methylation levels of cg00945507, cg05406101, cg10092957, cg14992108, cg18678121 were associated with favorable prognosis. On the contrary, high methylation level of cg13060154 was associated with poor survival (Supplementary Figures S1S–X).

On the basis of multivariate Cox regression, we developed the following risk model:

Risk score = −4.016 × cg00945507 + 1.117 × cg05406101−1.78 × cg10092957 + 1.655 × cg13060154−1.283 × cg14992108−1.136 × cg18678121 (Table 3).

**FIGURE 5:**
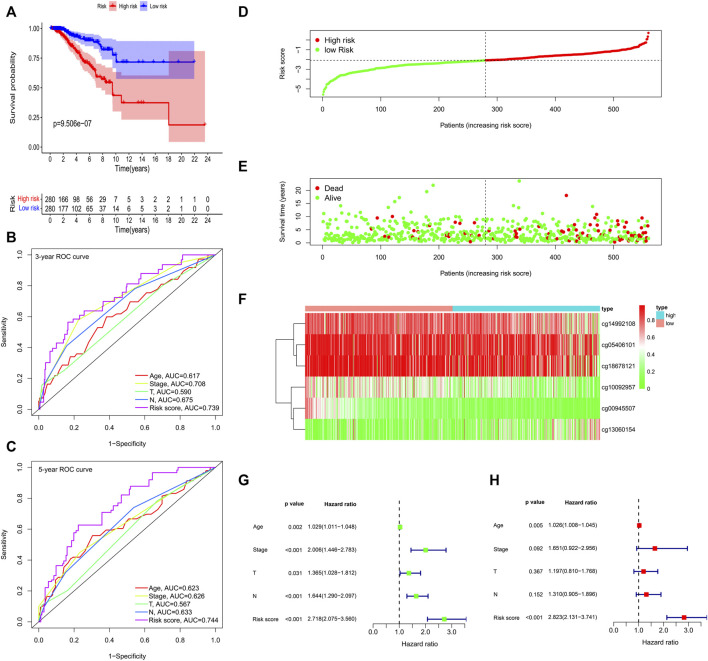
Methylation prognostic model assessment in 560 breast cancer samples. **(A)** Survival analysis between the high-risk and low-risk groups. **(B,C)** The time-dependent receiver operating characteristic (ROC) curves at 3 and 5 years. **(D)** The risk score distribution. **(E)** Survival status scatter plots. **(F)** Heatmap of the six signature CpG loci. **(G)** Univariate Cox regression analysis. **(H)** Multivariate Cox regression analysis.

According to the formula, we computed the risk score of each BC patient in the train group (*n* = 560) and assigned them into high-risk (*n* = 280) and low-risk groups (*n* = 280) with reference to the median risk score. The methylation levels of the six CpG loci between the high-risk group and low-risk group were showed in Supplementary Figures S2A. Kaplan-Meier curve indicated that the high-risk group had significantly poorer OS than the low-risk group (Figure 5A). To confirm the methylation risk score could effectively predict the BC patients’ prognosis, we plotted the ROC curve. Notably, we observed that the risk score had the highest prediction performance of prognosis compared with the conventional clinical features, with the 3-years and 5-years AUC values being 0.739 and 0.744 (Figures 5B,C). The relationship of methylation risk score, survival status and methylation levels of the six signature CpG loci was shown in Figures 5D–F. Univariate Cox analysis indicated that age, stage, T stage, N stage and risk score were significantly associated with OS. However, when they were introduced into multivariate Cox analysis, only age [hazard ratio, 1.026 (95% CI, 1.008–1.045), *p* = 0.005], and risk score [hazard ratio, 2.823 (95% CI, 2.131–3.741), *p* < 0.001] remained as independent prognostic predictors (Figures 5G,H). Similar prognostic significance was observed in the validation cohort (27k array dataset), with AUC values of 0.603 and 0.657 for 3 and 5 years, respectively (Supplementary Figures S2B–D).

### Identification of Differentially Expressed Genes From the Methylation High-Risk and Low-Risk Groups

**FIGURE 6:**
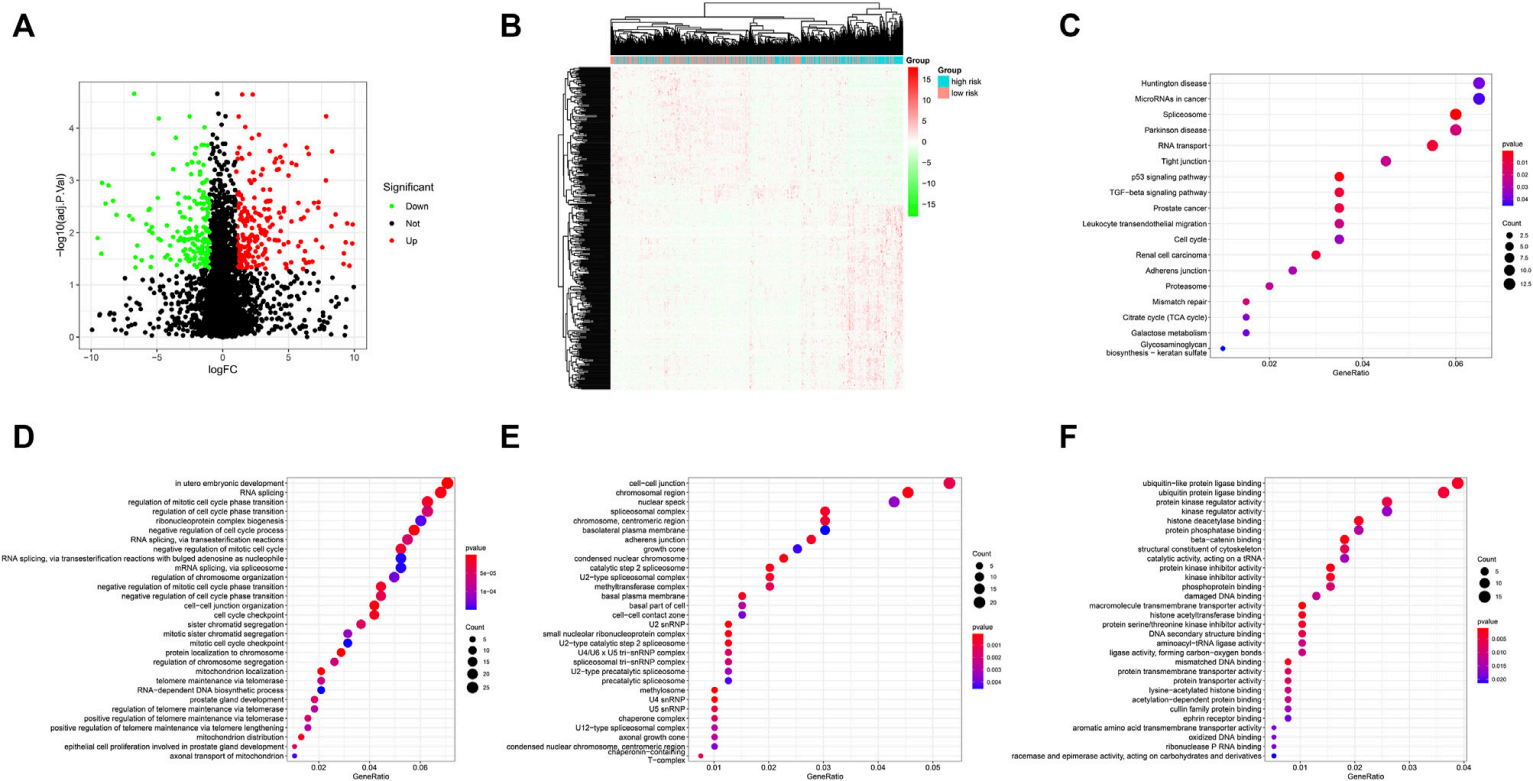
**(A)** Volcano plot of differentially expressed genes (DEGs); **(B)** Heatmap of the DEGs; **(C–F)** GO and KEGG enrichment analysis for DEGs. KEGG pathway **(C)**, BP **(D)**, CC **(E)**, and MF **(F)**.

Using the thresholds of adjusted *p* < 0.05 and | log2 (fold change) | >1, a total of 413 differentially expressed genes (DEGs) between methylation high-risk and low-risk groups were obtained. The volcano and heatmap visually displayed the DEGs (Figures 6A,B). To further investigate the biological characteristics of the DEGs, function and pathway annotations were performed. GO analysis indicated that these genes were involved in the regulation of cell cycle processes and mitotic cell cycle phase transition. KEGG analysis showed that these DEGs were mainly enriched in p53 and TGF-β signaling pathways (Figures 6C–F).

### Construction and Evaluation of Gene Prognostic Signature

To explore the prognostic values of the identified 413 DEGs, we extracted the expression of these DEGs from the transcriptomic profiles of 986 BC patients in the TCGA-BRCA database for subsequent analysis. Firstly, 50 DEGs significantly related with OS were selected by Kaplan-Meier analysis (*p* < 0.05). All of these prognostic genes were subjected to univariate Cox regression analysis and 13 of them with *p* < 0.05 were further identified as prognosis-associated genes (Supplementary Table S1). Ultimately, six prognosis-associated genes, namely *IRF2, KCNJ11, ZDHHC9, LRP11, PCMT1,* and *TMEM70*, were included in developing a gene prognostic signature by multivariate Cox regression analysis. Among them, four genes (*ZDHHC9, LRP11, PCMT1, TMEM70*) were related with poor survival and two genes (*IRF2, KCNJ11*) were associated with good survival (Supplementary Figures S3). The risk formula was as follows:

Risk score = −(0.06128 × *IRF2*) − (0.0342 × *KCNJ11*) + (0.0228 × *ZDHHC9*) + (0.01257 × *LRP11*) + (0.01082 × *PCMT1*) + (0.02917 × *TMEM70*).

**FIGURE 7:**
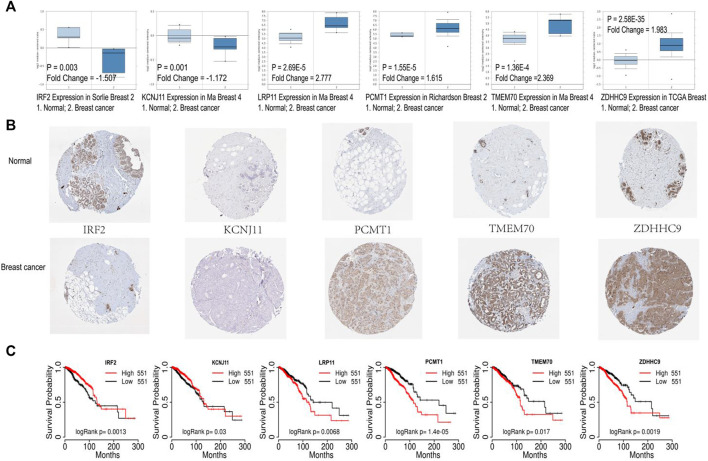
Validation of the six signature genes. **(A)** The expression of the six signature genes in breast cancer and normal tissue in Oncomine database. **(B)** The protein expression levels of the six prognostic genes in Human Protein Atlas database. **(C)** Survival analysis of the six prognostic genes based on TCGA portal.

Gene expression analysis of the six signature genes in the Oncomine database (https://www.oncomine.org) revealed that *ZDHHC9, LRP11, PCMT1, TMEM70* were highly expressed in breast cancer, and *IRF2, KCNJ11* were highly expressed in normal tissues (Figure 7A). Then we explored the protein levels of these six genes between breast cancer and normal tissues in the Human Protein Atlas database (Uhlen et al., 2010) (HPA: https://www.proteinatlas.org/humanproteome/pathology). In accordance with the gene expression levels, the protein levels of *ZDHHC9, PCMT1, TMEM70* were significantly higher in breast cancer, and the protein levels of *IRF2, KCNJ11* were higher in normal tissues (Figure 7B). Moreover, we further checked the prognostic values of our six genes in the public database TCGA portal (version 1.0) (http://tcgaportal.org/TCGA/Breast_TCGA_BRCA/process.php), and we found that *ZDHHC9, LRP11, PCMT1, TMEM70* were associated with poor prognosis, while *IRF2 and KCNJ11* were related with good prognosis of BC patients (Figure 7C).

### Association of Methylation Prognostic Model and Methylation-Based Gene Prognostic Model

Perhaps not surprisingly, a positive and significant correlation was observed between the two prognostic signatures, which was mainly reflected at the following aspects: firstly, a moderate correlation was found between the six signature CpG loci and six signature genes. Specifically, the expression of *IFR2* was positively related with the methylation of cg00945507, cg05406101, cg14992108, and cg18678121, above of which were all good prognostic factors, while *IFR2* expression was negatively related with the methylation of poor prognostic CpG locus: cg13060154; And *KCNJ11* expression was positively correlated with the methylation of favorable prognostic CpG loci: cg05406101 and cg10092957, and negatively related with the methylation of cg13060154; To the opposite of these two genes, *ZDHHC9* and *TMEM70* expression were negatively related with the methylation of cg05406101 and cg18678121, and positively associated with the methylation of cg13060154; Similarly, the expression of *PCMT1* was negatively correlated with the methylation of cg05406101, cg14992108, cg18678121; And *LRP11* expression was negatively related with the methylation of cg00945507, cg05406101, cg18678121 (Supplementary Figures S4A, Supplementary Figures S4I).

Subsequently, we examined the correlation between the six CpG loci and the gene risk score, and we observed that except for cg13060154 having the trend being positively correlated with the risk score, cg05406101 (R2 = −0.34, *p* < 0.001), cg18678121 (R2 = −0.28, *p* < 0.001), cg14992108 (R2 = −0.26, *p* < 0.001), cg00945507 (R2 = −0.26, *p* < 0.001), and cg10092957 (R2 = −0.11, *p* < 0.01) were all negatively correlated with the risk score (Supplementary Figures S4B–G, Supplementary Figures S4I **)**. Besides, we also explored the relationship between methylation risk score and gene risk score. Interestingly, we found that these two established risk scores were positively correlated with each other (R2 = 0.34, *p* < 0.001) (Supplementary Figures S4H,I).

### Evaluation and Validation of the Gene Prognostic Signature

**FIGURE 8:**
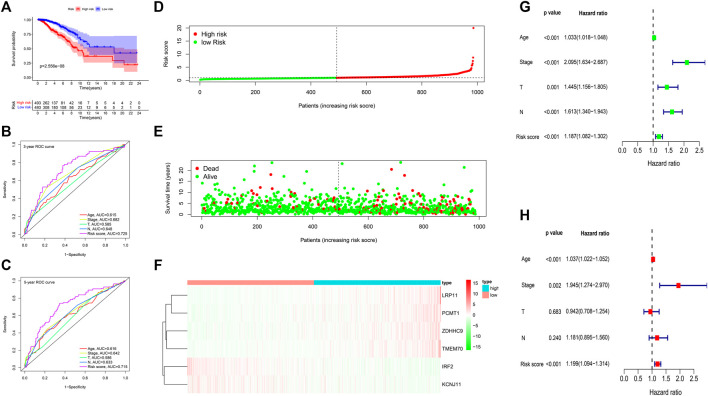
Gene prognostic signature assessment of 986 breast cancer samples. **(A)** Survival analysis between the high-risk and low-risk groups. **(B,C)** The time-dependent receiver operating characteristic (ROC) curves at 3 and 5 years. **(D)** The risk score distribution. **(E)** Survival status scatter plot. **(F)** Heatmap of the six prognostic genes. **(G)** Univariate Cox regression analysis. **(H)** Multivariate Cox regression analysis.

The expression of the six signature genes between the gene high-risk and low-risk groups was shown in Supplementary Figures S5A. Kaplan-Meier analysis showed that survival probability in the low-risk group was higher (Figure 8A). The AUC values of 3-years and 5-years OS were 0.725 and 0.715 (Figures 8B,C). The risk score distribution, the survival status, and the expression of the six genes of 986 BC patients were visualized in Figures 8D–F. Univariate and multivariate Cox regression analyses indicated that the risk score was associated with OS and could be an independent prognostic predictor, with univariate hazard ratio, 1.187 (95% CI, 1.082–1.302, *p* < 0.001), multivariate hazard ratio, 1.199 (95% CI, 1.094–1.314), *p* < 0.001, respectively (Figures 8G,H).

To confirm the prognostic value of the six-gene signature, we tested it with the validation subgroup comprising of 557 BC patients. The results were consistent with our previous findings. Specifically, the OS in the high-risk group was poorer (Supplementary Figures S5B), and the AUC values of 3 and 5 years were 0.735 and 0.696 (Supplementary Figures S5C). Univariate and multivariate COX regression analysis also showed that the risk score was an independent prognostic predictor of BC (Supplementary Figures S5D,E). Subsequently, the 986 BC patients’ dataset was randomly assigned into two test subgroups [test group one (*n* = 494) and test group two (*n* = 492)] which were with balanced baseline characteristics (Table 4), and both of them were used for validating the gene signature. The two subgroups could also distinguish the favorable OS patients from the poor OS patients (Supplementary Figures S5F,G). AUC values of 1 year, 3 years, 5 years were 0.711, 0.757, 0.721 in test group one, and 0.864, 0.733, 0.702 in test group two, respectively (Supplementary Figures S5I,J). Moreover, the external dataset GSE146558 further confirmed our gene prognostic signature. In high-risk group of GSE146558 dataset, patients were with poorer OS (Supplementary Figures S5H). And AUC value of 3 years was 0.634 (Supplementary Figures S5K).

**TABLE 4:** Clinical characteristics of the two validation subgroups from the dataset of 986 samples.

Covariates	Type	Total	Test group one	Test group two	*p* Value
Gender	Female	986 (100%)	492 (100%)	494 (100%)	0.9492
Age	≤65	716 (72.62%)	358 (72.76%)	358 (72.47%)	0.9742
—	>65	270 (27.38%)	134 (27.24%)	136 (27.53%)	—
Pathologic stage^a^	Stage I-II	727 (73.73%)	355 (72.15%)	372 (75.3%)	0.1261
—	Stage III-IV	239 (24.24%)	131 (26.63%)	108 (21.86%)	—
—	Unknow	20 (2.03%)	6 (1.22%)	14 (2.83%)	—
T stage^a^	T1-2	829 (84.08%)	404 (82.11%)	425 (86.03%)	0.1253
—	T3-4	154 (15.62%)	86 (17.48%)	68 (13.77%)	—
—	unknow	3 (0.3%)	2 (0.41%)	1 (0.2%)	—
M stage^a^	M0	811 (82.25%)	406 (82.52%)	405 (81.98%)	0.2694
—	M1	20 (2.03%)	7 (1.42%)	13 (2.63%)	—
—	Unknow	155 (15.72%)	79 (16.06%)	76 (15.38%)	—
N stage^a^	N0	451 (45.74%)	215 (43.7%)	236 (47.77%)	0.1875
—	N1-3	518 (52.54%)	270 (54.88%)	248 (50.2%)	—
—	Unknow	17 (1.72%)	7 (1.42%)	10 (2.02%)	—

a Staging according to Seventh Edition AJCC, Guidelines (Edge SB, Byrd DR, Compton CC, Fritz AG, Greene FL, Trotti A, eds. AJCC, Cancer Staging Manual. Seventh ed New York, NY: springer; 2010).

In addition, we confirmed the prognostic value of the six-gene prognostic signature in the subgroups of BC patients presented with different clinical features (age (<65 and≥65), T staging (T1-2 and T3-4), N staging [N0 and N1-3) and stage (stage I-II and stage III-IV)] (Supplementary Figure S6).

**FIGURE 9:**
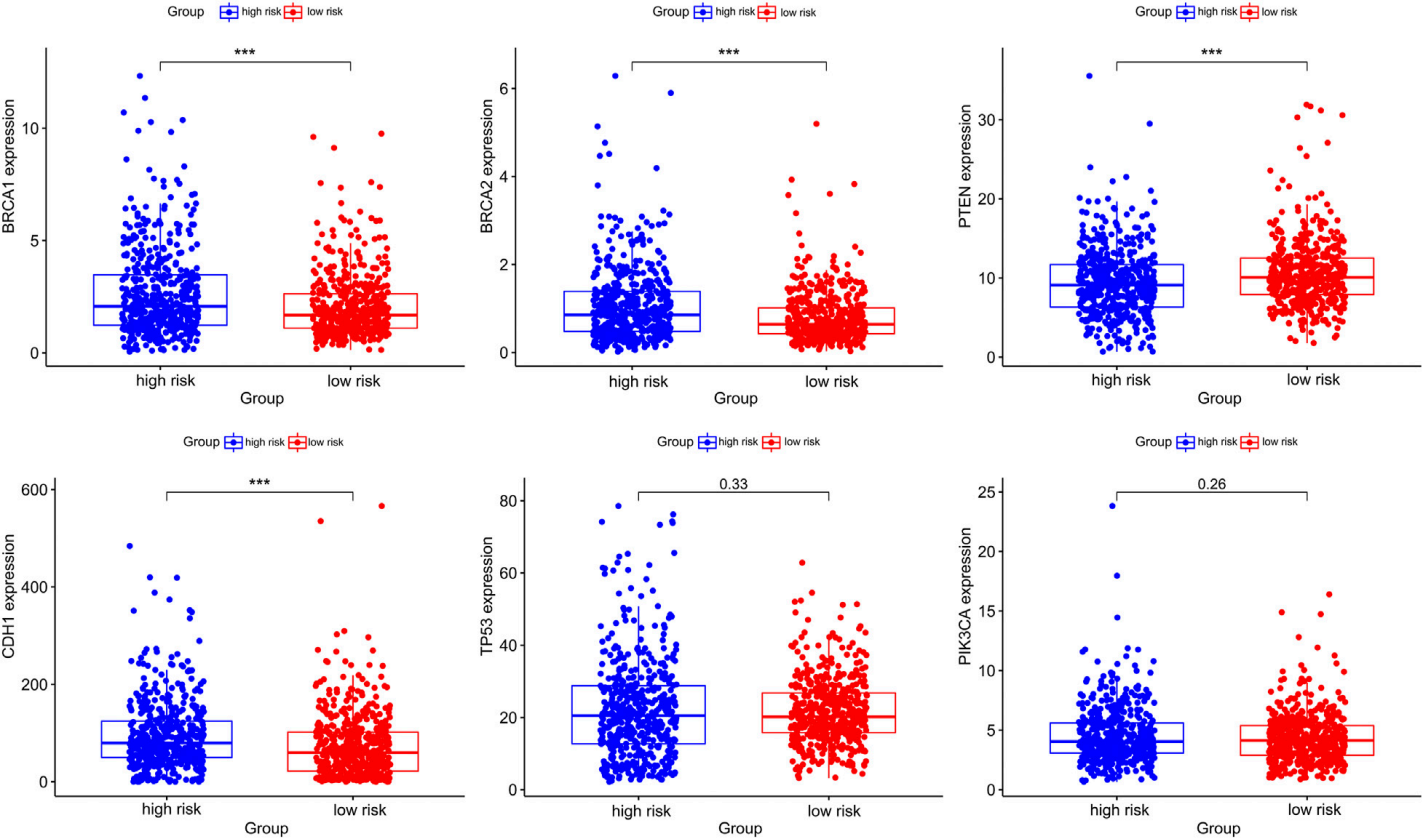
The differential expression of breast cancer-associated genes in gene high-risk and low-risk groups.

Considering the important roles of *BRCA1, BRCA2*, *CDH1, PTEN, TP53, PIK3CA* in BC, we also evaluated these gene expression between gene high-risk and low-risk groups, and observed that the expression of oncogenes such as *BRCA1, BRCA2* and *CDH1* were significantly higher in high-risk group. On the other hand, the expression of the tumor suppressor gene *PTEN* was significantly higher in low-risk group (Figure 9).

### Gene Set Enrichment Analysis

**FIGURE 10:**
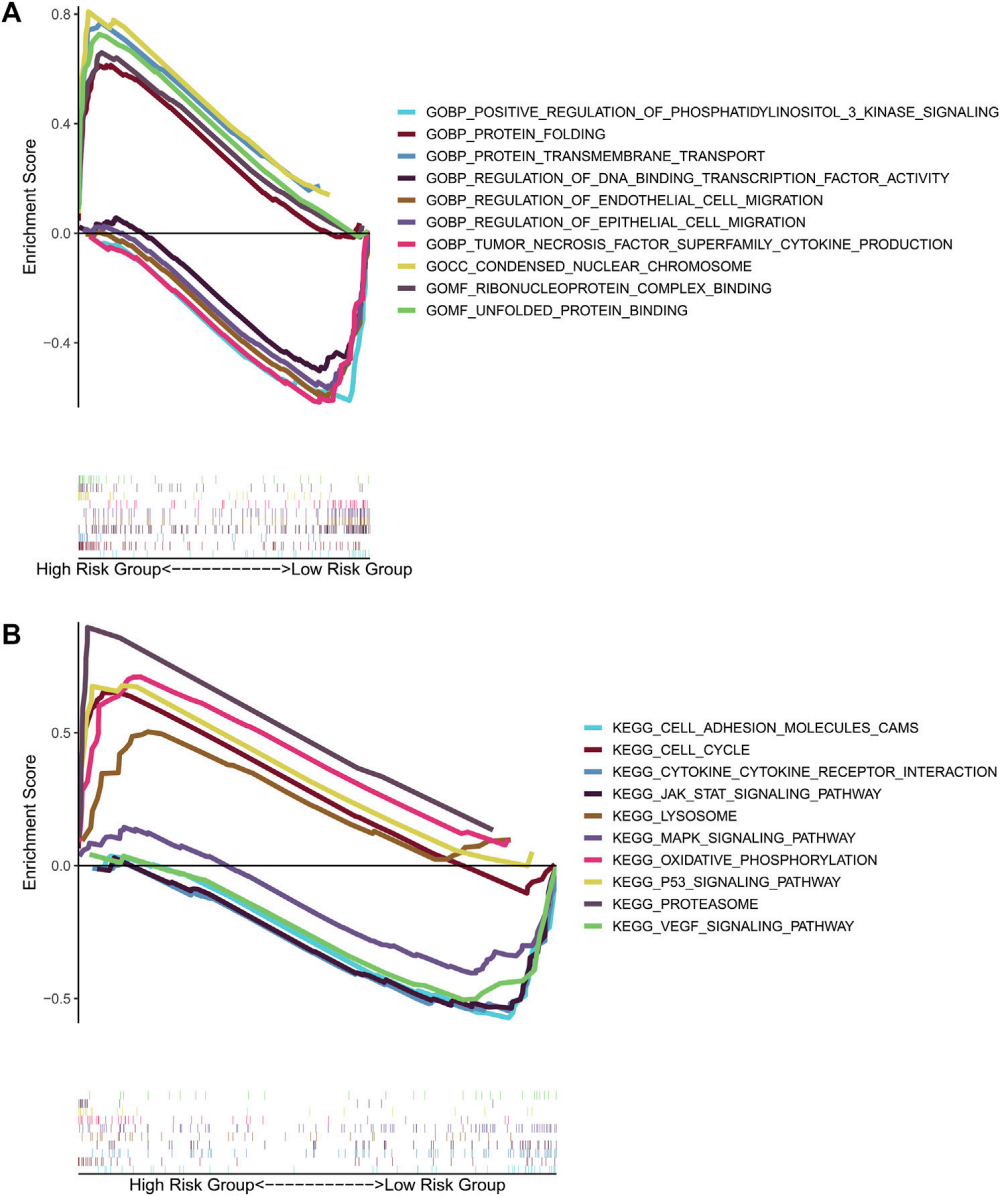
GSEA for the gene prognostic signature. **(A)** The significant enrichment of the top 5 tumor-related GO terms in high-risk group and low-risk group. **(B)** The significant enrichment of the top 5 tumor-related pathways in high-risk group and low-risk group.

To investigate potential functions and signaling pathways related to the six-prognosis signature, we performed Gene Set Enrichment Analysis (GSEA: http://www.gsea-msigdb.org/gsea/index.jsp). Notably, we found that more tumor-related GO terms and KEGG pathways were associated with low-risk group (Figure 10A). In detail, low-risk group was mainly associated with the function of regulating epithelial and endothelial cell migration, and high-risk group was related with nuclear chromosome condensing, protein folding. Pathway enrichment analysis indicated that JAK/STAT signaling pathway, cell adhesion molecule signaling pathway, VEGF signaling pathway, and MAPK signaling pathway were active in the low-risk group. On the other hand, P53 signaling pathway was active in the high-risk group (Figure 10B).

### Correlation Analysis of the Six Signature Genes and the Sensitivity of Anti-Tumor Drugs

**FIGURE 11:**
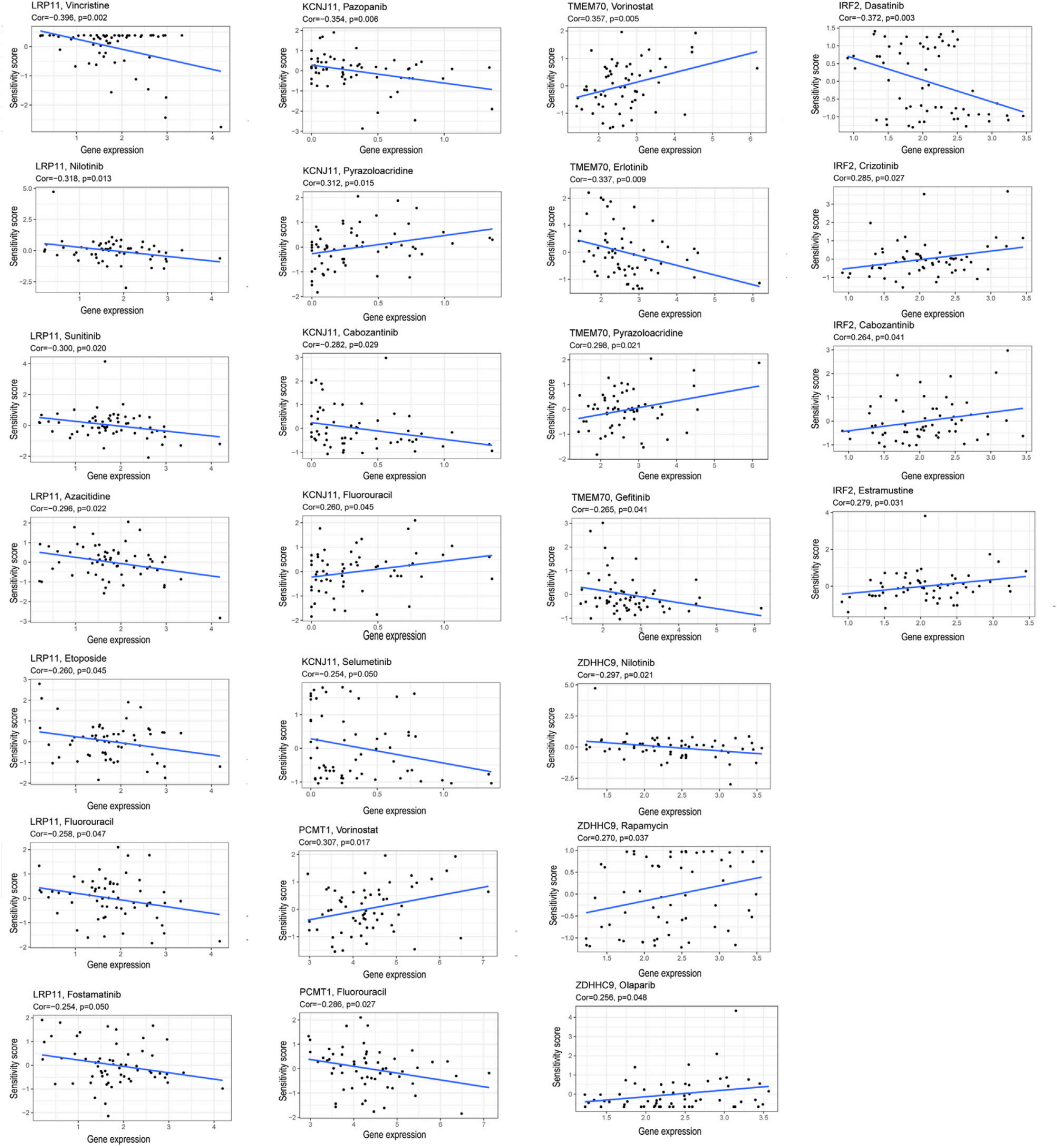
Correlation of the expression of the six prognostic genes and the sensitivity of anti-tumor drugs.

Correlation analysis between the expression of the six prognosis genes and the sensitivity of anti-tumor drugs was performed based on the CellMiner database (https://discover.nci.nih.gov/cellminer/), and the results indicated that our signature genes were moderately correlated with the response of some common anti-tumor drugs such as PARP inhibitor (Olaparlib), chemotherapy drugs (Fluorouracil, Decitabine, Oxaliplatin), which might imply potential value in anti-tumor therapy (Figure 11).

## Discussion

With the advent of next-generation sequencing, genome-wide DNA methylation profile analysis has become possible. Multiple studies have suggested that DNA methylation plays an important role in early detection, improved molecular classification, prognosis prediction of BC. Moreover, numerous studies have demonstrated that DNA methylation could regulate immune-related gene expression, thereby affecting the response of anti-tumor immunotherapy and BC patients’ prognosis. For examples, increasing researches have reported that the expression of immune genes such as CD3D, CD6, and HLA-A was found to be negatively correlated with DNA methylation, and was related with a better prognosis in BC (Győrffy et al., 2016). Potential targets for immunotherapy are still being explored. Recent studies have shown that immune cell infiltration might be a biomarker for immunotherapy. Importantly, the methylation of immune genes could also highly sensitively reflect the presence of tumor infiltrating lymphocytes. Thus, DNA methylation profiles could be used to predict the proportion of all kinds of immune cells in the tumor microenvironment (Győrffy et al., 2016), (Jeschke et al., 2015). Given the important role of DNA methylation, it is not surprising that a better understanding of the DNA methylation and the exploration of the interaction mechanism between genes and methylation are crucial for BC patients.

DNA methylation has a substantial impact on gene expression, and affects the prognosis of different subtypes of BC patients (44). In this study, we obtained six prognosis-related CpG loci, cg00945507, cg05406101, cg10092957, cg14992108, cg18678121, cg13060154, respectively targeting *SEC61G, RWDD2B, NCCRP1, SNTB1, SEC61A2, DAB2IP* genes. *SEC61G* was found to be overexpressed in BC and might co-amplify with epidermal growth factor receptor (EGFR) (Reis-Filho et al., 2006). Lu et al. reported that the expression of *SEC61G* in BC was negatively correlated with its promoter methylation (Lu et al., 2021). In our research, similar trend could be found that the expression of *SEC61G* was negatively related with the methylation of cg00945507. Moreover, the methylation level of *SEC61G* was positively correlated with the prognosis of patients with glioma (Liu et al., 2019a). Miwa et al. proved that *NCCRP1* transcription was inhibited by promoter hypermethylation in esophageal squamous cell carcinoma (Miwa et al., 2017). And high expression of *NCCRP1* in patients with pancreatic cancer was associated with a poor prognosis (Zuo et al., 2020). In our study, we observed that the expression of *NCCRP1* was negatively correlated with the methylation of cg10092957. *DAB2IP* was a candidate tumor suppressor gene and its expression down-regulation mechanism was mainly through the promoter hypermethylation (Qiu et al., 2007). Demethylation of *DAB2IP* gene weakened the EMT process and suppressed hepatocellular carcinoma growth (Liu et al., 2019b). However, we observed a weak positive correlation between *DAB2IP* expression and cg13060154 methylation in our study. Regrettably, studies on the correlation between the expression of *SEC61A2, RWDD2B, SNTB1* and DNA methylation in tumors were insufficient.

On the bias of the six prognostic CpG loci, we developed a methylation risk model that could accurately classify BC patients with different death risk. Subsequently, we identified 413 DEGs from the methylation high-risk and low-risk groups. Function enrichment analysis indicated that these DEGs were related with cell cycle checkpoint, ubiquitin-like protein ligase binding. KEGG pathway analysis showed these genes were mainly enriched in p53 signaling pathway, and TGF-β signaling pathway. The above functions and pathways were common and critical in tumor proliferation, invasion, and metastasis. And then, we further extracted the expression of the 413 DEGs from the transcriptomic profiles of 986 BC patients to evaluate the prognostic roles of these DEGs in TCGA-BRCA dataset. The prognostic value of individual genes or gene signatures has been extensively studied in cancers (Parker et al., 2009). Herein, we got a methylation-based gene prognostic signature using multivariate Cox analysis.

The six signature genes were composed of *IRF2, ZDHHC9, KCNJ11, LRP11, PCMT1,* and *TMEM70*. *IRF2,* a transcription factor in the interferon gamma signal transduction pathway, was different expression in breast cancer and normal tissues. Kriegsman et al. found that *IRF2,* which positively regulated the MHC class I pathway and negatively regulated PD-L1 expression, had good implications for immunotherapy and prognosis of BC (Kriegsman et al., 2019). *ZDHHC9,* one of risk genes of BC, was found to participate in palmitoylating PD-L1 to keep its protein stability, leading to immune escape. Inhibiting the *ZDHHC9* expression made breast cancer cells susceptible to T cell killing and inhibited tumor growth. Thus, ZDHHC9 could be a biomarker of immunotherapy response (Yang et al., 2019). *KCNJ11* played a key role in glucose-stimulated insulin secretion (Cook and Hales, 1984). It is well established that diabetes is closely related to a variety of tumors (Giovannucci et al., 2010), and the mortality is higher among women with longer diabetes duration in BC (Lega et al., 2018). Therefore, diabetes-related genes *KCNJ11* may also be a potential prognostic biomarker of BC. Yet, the relationship between *KCNJ11* and breast cancer has not been systematically reported. *PCMT1* has gradually been considered as a risk gene in tumors. Study demonstrated that BC patients with higher *PCMT1* expression had a poorer prognosis (Dong et al., 2021). Furthermore, the different expression of the six signature genes in mRNA and protein levels was validated in public databases.

The methylation-based gene signature could also distinguish BC patients with a significantly increased risk from those with a decreased risk. Moreover, correlation analysis showed that the methylation of the six signature CpGs were closely correlated with the expression of the six signature genes, and the established gene risk score was significantly positively correlated with the methylation risk score.

Multigene analysis has been popularized to predict the response of anti-tumor therapy and prognosis in BC. For instance, the EndoPredict score (12-gene molecular signature) has been used to predict the survival without distant recurrence up to 15 years after diagnosis. Recently, the 12-gene MS has also been proven to predict the response to neoadjuvant chemotherapy (NaCT) and neoendocrine therapy (NET) in HR+, her2- BC patients, with AUC values being 0.736 for NaCT, 0.726 for NET (Dubsky et al., 2020). Other widely used multigene assays involve Oncotype Dx, MammaPrint, and PAM50 which have been validated to predict the treatment response, recurrence, prognosis in BC patients. Among these multi-gene tests, MammaPrint has the best predictive performance (AUC = 0.88), following by Oncotype Dx (AUC = 0.76), PAM50 risk of relapse based on subtype (ROR-S) (AUC = 0.68) and the PAM50 risk of relapse based on subtype and proliferation (ROR-P) (AUC = 0.55) (Grimm and Mazurowski, 2020). Our study developed methylation and methylation-based prognostic signatures, both of which had excellent performance in predicting the prognosis of BC patients with 3-years, 5-years AUC values being 0.739, 0.744 for methylation signature, and 0.725, 0.715 for methylation-based gene signature. Three TCGA-BRCA subgroups were used to validate the gene prognostic signature and all of them showed powerful prediction effects with 3-years AUC values of 0.757, 0.735, 0.733, respectively. Moreover, the external dataset GSE146558 was also used to validate our gene prognostic signature. Due to small sample size (*n* = 106) and inter-dataset heterogeneity, we could not obtain a higher AUC value in the validation set, although the 3-years AUC value being 0.634 was still statistically significant.

Increasing researches reported that *CDH1, BRCA2,* and *BRCA1* were susceptibility genes for BC (Petridis et al., 2019) and around 60–70% of women with *BRCA1* or *BRCA2* gene mutations would be suffered with BC in her lifetime (Antoniou et al., 2003). Besides, *BRCA2* mutation carriers were more likely to develop brain metastases than non-carriers (Song et al., 2020). *PTEN* is a tumor suppressor gene in BC, and researches proved that lack or decrease of *PTEN* expression might be associated with poor prognosis in BC (Luen et al., 2018). Then we examined the expression alteration of these genes in gene high-risk and low-risk groups to understand the contribution of the gene signature to the carcinogenesis of BC. As expected, the expression of proto-oncogenes *BRCA1, BRCA2,* and *CDH1* was significantly higher in high-risk group. Conversely, expression of tumor suppressor gene *PTEN* was significantly higher in low-risk group.

There were some advantages of our study. First of all, we were the first one to discuss the prognostic roles of CpG loci in breast cancer, and constructed methylation-associated signatures. Secondly, these two prognostic signatures were positively correlated with each other and both of them could accurately discriminate breast cancer patients with different death risk. Besides, three subgroups of TCGA dataset and an external dataset GSE146558 were verified the prognostic value of our gene signature. Finally, the above results, together with risk gene expression verification, GSEA, drug sensitivity analysis, might provide novel treatment and prognosis biomarkers for breast cancer patients. We believe with the advent of the era of precision medicine, clinical trials could be designed using gene signature-based risk scores to select the patients most likely to develop poor prognosis in which to develop novel or more intensive postoperative therapies in future.

One major limitation for our study was that data in our study was downloaded from the public databases, the mechanism of the six signature genes and six signature CpGs affecting the occurrence and development of breast cancer still needs to be further verified by vivo and vitro experiments. Even, prospective clinical trials are needed to check the prognostic values of these two signatures.

## Conclusion

Taken together, we proposed two methylation-related prognostic signatures. These two signatures were significantly positively correlated with each other and both of them could predict the prognosis of BC patients more accurately than traditional clinical predictors. Importantly, the six key genes (*IRF2, ZDHHC9, KCNJ11, LRP11, PCMT1, TMEM70*) of gene prognostic signature may act as potential prognostic biomarkers and therapeutic targets.

## Data Availability

The original contributions presented in the study are included in the article/Supplementary Material, further inquiries can be directed to the corresponding authors.

## Supplementary Material

Supplementary Figure S1
**(A–F)** The correlation between the six signature CpGs and their target genes. **(A)** DAB2IP, **(B)** NCCRP1, **(C)** RWDD2B, **(D)** SEC61A2, **(E)** SEC61G, **(F)** SNTB1; **(G–L)** Differential expression of target genes in the hypermethylation and hypomethylation of the six signature CpGs. **(G)** DAB2IP, **(H)** NCCRP1, **(I)** RWDD2B, **(J)** SEC61A2, **(K)** SEC61G, **(L)** SNTB1; **(M–R)** The methylated levels of the CpGs of the target genes in breast cancer and normal tissue in MethSurv. **(M)** cg13060154, **(N)** cg14992108, **(O)** cg18678121, **(P)** cg10092957, **(Q)** cg05406101, **(R)** cg00945507; **(S–X)** The survival analysis of the high- and low-methylation levels of the CpGs of target genes in MethSurv. **(S)** SEC61A2, **(T)** RWDD2B, **(U)** DAB2IP, **(V)** SEC61G, **(W)** NCCRP1, **(X)** SNTB1.

Supplementary Figure S2
**(A)** The methylated levels of the six signature CpGs between methylation high-risk and low-risk groups of 560 breast cancer samples. **(B–D)** Methylation prognostic model assessment in the testing group of 278 breast cancer samples. **(B)** Survival analysis between the high-risk and low-risk groups. **(C,D)** The time-dependent receiver operating characteristic (ROC) curves at 3 and 5 years.

Supplementary Figure S3 Survival analysis of the six signature genes.

Supplementary Figure S4
**(A–I)** The association of the methylation prognostic model and the gene prognostic signature. **(A)** The correlation of the six signature genes and the six signature CpGs. **(B–G)** The correlation between the β value of the six CpGs and the gene risk scores. **(H)** The correlation between methylation risk scores and gene risk scores. **(I)** The correlation of the methylation prognostic model and gene prognostic model.

Supplementary Figure S5
**(A–I)** **(A)** The expression of the 6 signature genes between gene high-risk and low-risk groups of 986 breast cancer samples; **(B–E)** Validation of the gene prognostic signature in a BRCA-TCGA subgroups composed of 557 samples, **(B)** Survival analysis, **(C)** ROC curve, (D) Univariate Cox regression analysis, (e) Multivariate Cox regression analysis; **(F–H)** Survival analysis in the other two BRCA-TCGA subgroups and the GSE146558 dataset, **(F)** survival analysis in the subgroups composed of 494 samples, **(G)** Survival analysis in the subgroups composed of 492 samples, **(H)** Survival analysis in the GSE146558 dataset; **(I–K)** ROC curves in the other two BRCA-TCGA subgroups and the GSE146558 dataset, **(I)** ROC curve in the subgroups composed of 494 samples, **(J)** ROC curve the subgroups composed of 492 samples, **(K)** ROC curve in the GSE146558 dataset.

Supplementary Figure S6 The overall survival analysis of breast cancer patients with different clinical characteristics in gene high-risk and low-risk groups.

## References

[B1] Achrol A. S. Rennert R. C. Anders C. Soffietti R. Ahluwalia M. S. Nayak L. (2019). Brain Metastases. Nat. Rev. Dis. Primers 5 (1), 5. 10.1038/s41572-018-0055-y 30655533

[B2] Allemani C. Matsuda T. Di Carlo V. Harewood R. Matz M. Nikšić M. (2018). Global Surveillance of Trends in Cancer Survival 2000-14 (CONCORD-3): Analysis of Individual Records for 37 513 025 Patients Diagnosed with One of 18 Cancers from 322 Population-Based Registries in 71 Countries. Lancet 391 (10125), 1023–1075. 10.1016/S0140-6736(17)33326-3 29395269 PMC5879496

[B3] Antoniou A. Pharoah P. D. P. Narod S. Risch H. A. Eyfjord J. E. Hopper J. L. (2003). Average Risks of Breast and Ovarian Cancer Associated with BRCA1 or BRCA2 Mutations Detected in Case Series Unselected for Family History: a Combined Analysis of 22 Studies. Am. J. Hum. Genet. 72 (5), 1117–1130. 10.1086/375033 12677558 PMC1180265

[B4] Chen Q. Hu L. Huang D. Chen K. Qiu X. Qiu B. (2020). Six-lncRNA Immune Prognostic Signature for Cervical Cancer. Front. Genet. 11, 533628. 10.3389/fgene.2020.533628 33173530 PMC7591729

[B5] Chen X. Zhang J. Ruan W. Huang M. Wang C. Wang H. (2020). Urine DNA Methylation Assay Enables Early Detection and Recurrence Monitoring for Bladder Cancer. J. Clin. Invest. 130 (12), 6278–6289. 10.1172/jci139597 32817589 PMC7685755

[B6] Cook D. L. Hales N. (1984). Intracellular ATP Directly Blocks K+ Channels in Pancreatic B-Cells. Nature 311 (5983), 271–273. 10.1038/311271a0 6090930

[B7] DeSantis C. E. Ma J. Gaudet M. M. Newman L. A. Miller K. D. Goding Sauer A. (2019). Breast Cancer Statistics, 2019. CA A. Cancer J. Clin. 69 (6), 438–451. 10.3322/caac.21583 31577379

[B8] Díez-Villanueva A. Mallona I. Peinado M. A. (2015). Wanderer, an Interactive Viewer to Explore DNA Methylation and Gene Expression Data in Human Cancer. Epigenetics & Chromatin 8, 22. 10.1186/s13072-015-0014-8 26113876 PMC4480445

[B9] Ding W. Chen G. Shi T. (2019). Integrative Analysis Identifies Potential DNA Methylation Biomarkers for Pan-Cancer Diagnosis and Prognosis. Epigenetics 14 (1), 67–80. 10.1080/15592294.2019.1568178 30696380 PMC6380428

[B10] Dong L.-M. Zhang X.-L. Mao M.-H. Li Y.-P. Zhang X.-Y. Xue D.-W. (2021). LINC00511/miRNA-143-3p Modulates Apoptosis and Malignant Phenotype of Bladder Carcinoma Cells via PCMT1. Front. Cel Dev. Biol. 9, 650999. 10.3389/fcell.2021.650999 PMC8063617 33898446

[B11] Dubsky P. C. Singer C. F. Egle D. Wette V. Petru E. Balic M. (2020). The EndoPredict Score Predicts Response to Neoadjuvant Chemotherapy and Neoendocrine Therapy in Hormone Receptor-Positive, Human Epidermal Growth Factor Receptor 2-negative Breast Cancer Patients from the ABCSG-34 Trial. Eur. J. Cancer 134, 99–106. 10.1016/j.ejca.2020.04.020 32502940

[B12] FitzGerald M. G. Marsh D. J. Wahrer D. Bell D. Caron S. Shannon K. E. (1998). Germline Mutations in PTEN Are an Infrequent Cause of Genetic Predisposition to Breast Cancer. Oncogene 17 (6), 727–731. 10.1038/sj.onc.1201984 9715274

[B13] Freddie B. Jacques F. Isabelle S. Ahmedin J. (2020). Erratum: Global Cancer Statistics 2018: GLOBOCAN Estimates of Incidence and Mortality Worldwide for 36 Cancers in 185 Countries. CA Cancer J. Clin. 70 (4), 313. 10.3322/caac.21609 32767693

[B14] Giovannucci E. Harlan D. M. Archer M. C. Bergenstal R. M. Gapstur S. M. Habel L. A. (2010). Diabetes and Cancer: a Consensus Report. Diabetes Care 33 (7), 1674–1685. 10.2337/dc10-0666 20587728 PMC2890380

[B15] Goldman M. J. Craft B. Hastie M. Repečka K. McDade F. Kamath A. (2020). Visualizing and Interpreting Cancer Genomics Data via the Xena Platform. Nat. Biotechnol. 38 (6), 675–678. 10.1038/s41587-020-0546-8 32444850 PMC7386072

[B16] Grimm L. J. Mazurowski M. A. (2020). Breast Cancer Radiogenomics: Current Status and Future Directions. Acad. Radiol. 27 (1), 39–46. 10.1016/j.acra.2019.09.012 31818385

[B17] Győrffy B. Bottai G. Fleischer T. Munkácsy G. Budczies J. Paladini L. (2016). Aberrant DNA Methylation Impacts Gene Expression and Prognosis in Breast Cancer Subtypes. Int. J. Cancer 138 (1), 87–97. 10.1002/ijc.29684 26174627

[B18] Huang K. K. Ramnarayanan K. Zhu F. Srivastava S. Xu C. Tan A. L. K. (2018). Genomic and Epigenomic Profiling of High-Risk Intestinal Metaplasia Reveals Molecular Determinants of Progression to Gastric Cancer. Cancer Cell 33 (1), 137–150. 10.1016/j.ccell.2017.11.018 29290541

[B19] Jeschke J. Collignon E. Fuks F. (2015). DNA Methylome Profiling beyond Promoters - Taking an Epigenetic Snapshot of the Breast Tumor Microenvironment. FEBS J. 282 (9), 1801–1814. 10.1111/febs.13125 25331982

[B20] Kanehisa M. Furumichi M. Tanabe M. Sato Y. Morishima K. (2017). KEGG: New Perspectives on Genomes, Pathways, Diseases and Drugs. Nucleic Acids Res. 45 (D1), D353–D361. 10.1093/nar/gkw1092 27899662 PMC5210567

[B21] King M.-C. Marks J. H. Mandell J. B. Grp N. Y. B. C. S. (2003). Breast and Ovarian Cancer Risks Due to Inherited Mutations in BRCA1 and BRCA2. Science 302 (5645), 643–646. 10.1126/science.1088759 14576434

[B22] Kresovich J. K. Xu Z. O’Brien K. M. Weinberg C. R. Sandler D. P. Taylor J. A. (2019). Methylation-Based Biological Age and Breast Cancer Risk. J. Natl. Cancer Inst. 111 (10), 1051–1058. 10.1093/jnci/djz020 30794318 PMC6792078

[B23] Kriegsman B. A. Vangala P. Chen B. J. Meraner P. Brass A. L. Garber M. (2019). Frequent Loss of IRF2 in Cancers Leads to Immune Evasion through Decreased MHC Class I Antigen Presentation and Increased PD-L1 Expression. J.I. 203 (7), 1999–2010. 10.4049/jimmunol.1900475 PMC6761035 31471524

[B24] Leek J. T. Johnson W. E. Parker H. S. Jaffe A. E. Storey J. D. (2012). The Sva Package for Removing Batch Effects and Other Unwanted Variation in High-Throughput Experiments. Bioinformatics 28 (6), 882–883. 10.1093/bioinformatics/bts034 22257669 PMC3307112

[B25] Lega I. C. Austin P. C. Fischer H. D. Fung K. Krzyzanowska M. K. Amir E. (2018). The Impact of Diabetes on Breast Cancer Treatments and Outcomes: A Population-Based Study. Dia Care 41 (4), 755–761. 10.2337/dc17-2012 29351960

[B26] Liang W. Zhao Y. Huang W. Gao Y. Xu W. Tao J. (2019). Non-invasive Diagnosis of Early-Stage Lung Cancer Using High-Throughput Targeted DNA Methylation Sequencing of Circulating Tumor DNA (ctDNA). Theranostics 9 (7), 2056–2070. 10.7150/thno.28119 31037156 PMC6485294

[B27] Liu B. Liu J. Liao Y. Jin C. Zhang Z. Zhao J. (2019). Identification of SEC61G as a Novel Prognostic Marker for Predicting Survival and Response to Therapies in Patients with Glioblastoma. Med. Sci. Monit. 25, 3624–3635. 10.12659/msm.916648 31094363 PMC6536036

[B28] Liu Z. Yu Y. Huang Z. Kong Y. Hu X. Xiao W. (2019). CircRNA-5692 Inhibits the Progression of Hepatocellular Carcinoma by Sponging miR-328-5p to Enhance DAB2IP Expression. Cell Death Dis 10 (12), 900. 10.1038/s41419-019-2089-9 31776329 PMC6881381

[B29] Lu T. Chen Y. Gong X. Guo Q. Lin C. Luo Q. (2021). SEC61G Overexpression and DNA Amplification Correlates with Prognosis and Immune Cell Infiltration in Head and Neck Squamous Cell Carcinoma. Cancer Med. 10(21), 7847–7862. 34590792 10.1002/cam4.4301 PMC8559468

[B30] Luen S. J. Asher R. Lee C. K. Savas P. Kammler R. Dell’Orto P. (2018). Association of Somatic Driver Alterations with Prognosis in Postmenopausal, Hormone Receptor-Positive, HER2-Negative Early Breast Cancer. JAMA Oncol. 4 (10), 1335–1343. 10.1001/jamaoncol.2018.1778 29902286 PMC6233777

[B31] Luo S. Chen J. Mo X. (2016). The Association of PTEN Hypermethylation and Breast Cancer: a Meta-Analysis. Ott Vol. 9, 5643–5650. 10.2147/ott.s111684 PMC5026181 27672335

[B32] Martin A. M. Cagney D. N. Catalano P. J. Warren L. E. Bellon J. R. Punglia R. S. (2017). Brain Metastases in Newly Diagnosed Breast Cancer. JAMA Oncol. 3 (8), 1069–1077. 10.1001/jamaoncol.2017.0001 28301662 PMC5824221

[B33] Mittendorf E. A. Zhang H. Barrios C. H. Saji S. Jung K. H. Hegg R. (2020). Neoadjuvant Atezolizumab in Combination with Sequential Nab-Paclitaxel and Anthracycline-Based Chemotherapy versus Placebo and Chemotherapy in Patients with Early-Stage Triple-Negative Breast Cancer (IMpassion031): a Randomised, Double-Blind, Phase 3 Trial. The Lancet 396 (10257), 1090–1100. 10.1016/s0140-6736(20)31953-x 32966830

[B34] Miwa T. Kanda M. Koike M. Iwata N. Tanaka H. Umeda S. (2017). Identification of NCCRP1 as an Epigenetically Regulated Tumor Suppressor and Biomarker for Malignant Phenotypes of Squamous Cell Carcinoma of the Esophagus. Oncol. Lett. 14 (4), 4822–4828. 10.3892/ol.2017.6753 29085486 PMC5649603

[B35] Modhukur V. Iljasenko T. Metsalu T. Lokk K. Laisk-Podar T. Vilo J. (2018). MethSurv: A Web Tool to Perform Multivariable Survival Analysis Using DNA Methylation Data. Epigenomics 10 (3), 277–288. 10.2217/epi-2017-0118 29264942

[B36] Nakao M. (2001). Epigenetics: Interaction of DNA Methylation and Chromatin. Gene 278 (1-2), 25–31. 10.1016/s0378-1119(01)00721-1 11707319

[B37] Park S. Y. Kwon H. J. Choi Y. Lee H. E. Kim S.-W. Kim J. H. (2012). Distinct Patterns of Promoter CpG Island Methylation of Breast Cancer Subtypes Are Associated with Stem Cell Phenotypes. Mod. Pathol. 25 (2), 185–196. 10.1038/modpathol.2011.160 22037257

[B38] Parker J. S. Mullins M. Cheang M. C. U. Leung S. Voduc D. Vickery T. (2009). Supervised Risk Predictor of Breast Cancer Based on Intrinsic Subtypes. Jco 27 (8), 1160–1167. 10.1200/jco.2008.18.1370 PMC2667820 19204204

[B39] Pasculli B. Barbano R. Parrella P. (2018). Epigenetics of Breast Cancer: Biology and Clinical Implication in the Era of Precision Medicine. Semin. Cancer Biol. 51, 22–35. 10.1016/j.semcancer.2018.01.007 29339244

[B40] Petridis C. Arora I. Shah V. Moss C. L. Mera A. Clifford A. (2019). Frequency of Pathogenic Germline Variants in CDH1, BRCA2, CHEK2, PALB2, BRCA1, and TP53 in Sporadic Lobular Breast Cancer. Cancer Epidemiol. Biomarkers Prev. 28 (7), 1162–1168. 10.1158/1055-9965.epi-18-1102 31263054

[B41] Pharoah P. D. P. Guilford P. Caldas C. International Gastric Cancer Linkage C. (2001). Incidence of Gastric Cancer and Breast Cancer in CDH1 (E-Cadherin) Mutation Carriers from Hereditary Diffuse Gastric Cancer Families. Gastroenterology 121 (6), 1348–1353. 10.1053/gast.2001.29611 11729114

[B42] Qiu G.-H. Xie H. Wheelhouse N. Harrison D. Chen G. G. Salto-Tellez M. (2007). Differential Expression of hDAB2IPA and hDAB2IPB in normal Tissues and Promoter Methylation of hDAB2IPA in Hepatocellular Carcinoma. J. Hepatol. 46 (4), 655–663. 10.1016/j.jhep.2006.11.012 17258345

[B43] Redig A. J. McAllister S. S. (2013). Breast Cancer as a Systemic Disease: a View of Metastasis. J. Intern. Med. 274 (2), 113–126. 10.1111/joim.12084 23844915 PMC3711134

[B44] Reinhold W. C. Varma S. Sunshine M. Rajapakse V. Luna A. Kohn K. W. (2017). The NCI-60 Methylome and its Integration into CellMiner. Cancer Res. 77 (3), 601–612. 10.1158/0008-5472.can-16-0655 27923837 PMC5290136

[B45] Reis-Filho J. Pinheiro C. Lambros M. Milanezi F. Carvalho S. Savage K. (2006). EGFR Amplification and Lack of Activating Mutations in Metaplastic Breast Carcinomas. J. Pathol. 209 (4), 445–453. 10.1002/path.2004 16739104

[B46] Schmid P. Cortes J. Pusztai L. McArthur H. Kümmel S. Bergh J. (2020). Pembrolizumab for Early Triple-Negative Breast Cancer. N. Engl. J. Med. 382 (9), 810–821. 10.1056/nejmoa1910549 32101663

[B47] Shen S. Wang G. Zhang R. Zhao Y. Yu H. Wei Y. (2019). Development and Validation of an Immune Gene-Set Based Prognostic Signature in Ovarian Cancer. EBioMedicine 40, 318–326. 10.1016/j.ebiom.2018.12.054 30594555 PMC6412087

[B48] Siegel R. L. Miller K. D. Jemal A. (2017). Cancer Statistics, 2017. CA: A Cancer J. Clinicians 67 (1), 7–30. 10.3322/caac.21387 28055103

[B49] Song Y. Barry W. T. Seah D. S. Tung N. M. Garber J. E. Lin N. U. (2020). Patterns of Recurrence and Metastasis in BRCA1/BRCA2 ‐associated Breast Cancers. Cancer 126 (2), 271–280. 10.1002/cncr.32540 31581314 PMC7003745

[B50] Stefansson O. A. Esteller M. (2013). Epigenetic Modifications in Breast Cancer and Their Role in Personalized Medicine. Am. J. Pathol. 183 (4), 1052–1063. 10.1016/j.ajpath.2013.04.033 23899662

[B51] Stefansson O. A. Moran S. Gomez A. Sayols S. Arribas-Jorba C. Sandoval J. (2015). A DNA Methylation-Based Definition of Biologically Distinct Breast Cancer Subtypes. Mol. Oncol. 9 (3), 555–568. 10.1016/j.molonc.2014.10.012 25468711 PMC5528700

[B52] Strahl B. D. Allis C. D. (2000). The Language of Covalent Histone Modifications. Nature 403 (6765), 41–45. 10.1038/47412 10638745

[B53] Suijkerbuijk K. P. M. Fackler M. J. Sukumar S. van Gils C. H. van Laar T. van der Wall E. (2008). Methylation Is Less Abundant in BRCA1-Associated Compared with Sporadic Breast Cancer. Ann. Oncol. 19 (11), 1870–1874. 10.1093/annonc/mdn409 18647968 PMC2733079

[B54] Tulotta C. Ottewell P. (2018). The Role of IL-1B in Breast Cancer Bone Metastasis. Endocr. Relat. Cancer 25 (7), R421–R434. 10.1530/erc-17-0309 29760166 PMC5987176

[B65] Uhlen M. Oksvold P. Fagerberg L. Lundberg E. Jonasson K. Forsberg M. (2010). Towards a Knowledge-Based Human Protein Atlas. Nat. Biotechnol. 28 (12), 1248–1250. 21139605 10.1038/nbt1210-1248

[B55] Walsh T. Casadei S. Coats K. H. Swisher E. Stray S. M. Higgins J. (2006). Spectrum of Mutations in BRCA1, BRCA2, CHEK2, and TP53 in Families at High Risk of Breast Cancer. Jama 295 (12), 1379–1388. 10.1001/jama.295.12.1379 16551709

[B56] Wang S. Gribskov M. Hazbun T. R. Pascuzzi P. E. Companion Cell. Miner. (2016). CellMiner Companion: an Interactive Web Application to Explore CellMiner NCI-60 Data. Bioinformatics 32 (15), 2399–2401. 10.1093/bioinformatics/btw162 27153600

[B57] Wilkerson M. D. Hayes D. N. (2010). ConsensusClusterPlus: a Class Discovery Tool with Confidence Assessments and Item Tracking. Bioinformatics 26 (12), 1572–1573. 10.1093/bioinformatics/btq170 20427518 PMC2881355

[B58] Woo H. D. Fernandez-Jimenez N. Ghantous A. Degli Esposti D. Cuenin C. Cahais V. (2018). Genome-wide Profiling of normal Gastric Mucosa identifiesHelicobacter Pylori- and Cancer-Associated DNA Methylome Changes. Int. J. Cancer 143 (3), 597–609. 10.1002/ijc.31381 29574700

[B59] Xu Z. Sandler D. P. Taylor J. A. (2020). Blood DNA Methylation and Breast Cancer: A Prospective Case-Cohort Analysis in the Sister Study. J. Natl. Cancer Inst. 112 (1), 87–94. 10.1093/jnci/djz065 30989176 PMC7489106

[B60] Yang I. V. Schwartz D. A. (2011). Epigenetic Control of Gene Expression in the Lung. Am. J. Respir. Crit. Care Med. 183 (10), 1295–1301. 10.1164/rccm.201010-1579pp 21596832 PMC3114059

[B61] Yang Y. Hsu J.-M. Sun L. Chan L.-C. Li C.-W. Hsu J. L. (2019). Palmitoylation Stabilizes PD-L1 to Promote Breast Tumor Growth. Cell Res 29 (1), 83–86. 10.1038/s41422-018-0124-5 30514902 PMC6318320

[B62] Yu G. Wang L.-G. Han Y. He Q.-Y. (2012). clusterProfiler: an R Package for Comparing Biological Themes Among Gene Clusters. OMICS: A J. Integr. Biol. 16 (5), 284–287. 10.1089/omi.2011.0118 PMC3339379 22455463

[B63] Zhang Z. (2016). Semi-parametric Regression Model for Survival Data: Graphical Visualization with R. Ann. Transl. Med. 4 (23), 461. 10.21037/atm.2016.08.61 28090517 PMC5220043

[B64] Zuo H. Chen L. Li N. Song Q. (2020). Identification of a Ubiquitination-Related Gene Risk Model for Predicting Survival in Patients with Pancreatic Cancer. Front. Genet. 11, 612196. 10.3389/fgene.2020.612196 33414811 PMC7782244

